# Supplementary material for: Protein structure generation via folding diffusion
Source: Nat Commun. 2024 Feb 5;15:1059. doi: 10.1038/s41467-024-45051-2 (PMC10844308; doi:10.1038/s41467-024-45051-2)
Supplement: Supplementary file 1 — Supplementary Information [file 41467_2024_45051_MOESM1_ESM.pdf]

Supplementary Information for:

Protein structure generation via folding diffusion

Kevin E. Wu[1,2,3,†], Kevin K. Yang[4], Rianne van den Berg[5], Sarah Alamdari[4], James Y. Zou[1,3], Alex X. Lu[4], Ava P. Amini[4,*]

[1]Department of Computer Science, Stanford University, Stanford, CA, USA

[2]Center for Personal Dynamic Regulomes, Stanford University, Stanford, CA, USA

[3]Department of Biomedical Data Science, Stanford University School of Medicine, Stanford, CA, USA

[4]Microsoft Research, Cambridge, MA, USA

[5]Microsoft Research, Amsterdam, Netherlands

[†]Work done principally during an internship at Microsoft Research

[*]Corresponding author; email `ava.amini@microsoft.com`

Angular representation of proteins

Choice of angles for protein representation

A protein backbone structure can be fully specified by a total of 9 values per residue: 3 bond distances, 3 bond angles, and 3 dihedral torsional angles. The three bond angles and dihedrals are described in Table 1, and the three bond distances correspond to $N_i \rightarrow C\alpha_i$, $C\alpha_i \rightarrow C_i$, and $C_i \rightarrow N_{i+1}$ where i denotes residue index. These 9 values enable a protein backbone to be losslessly converted from Cartesian to internal angle representation, and vice versa. To determine which subset of values to use to formulate proteins in our model, we take a set of experimentally profiled proteins and translate their coordinates from Cartesian to internal angles and distances and back, measuring the TM score between the initial and reconstructed structures. When excluding an angle or distance, we fix all corresponding values to the mean. The reconstruction TM scores of various combinations of values is illustrated in Figure S1. Of these 9 values, the three bond distances are the least important for reliably reconstructing a structure from Cartesian coordinates to the inter-residue representation and back; they can usually be replaced with constant average values without much impact on the recovered structure. In comparison, removing even two bond angles with relatively little variance (θ_2, θ_3) results in a large loss in reconstruction TM score (third bar). Removing all bond angles and retaining only dihedrals (ϕ, ψ, ω) results in only about half of proteins being able to be reconstructed (last bar). As a side note, prior works performing angle-based structure generation only used the ϕ, ψ dihedrals [25], which our analysis shows are overly lossy when specifying a global structure; this may have limited the success of these prior methods. Thus, we model the three dihedrals and the three bond angles (second bar in Figure S1); this simplifies our prediction problem to use only periodic angular values (instead of a mixture of angular and real values) without a substantial loss in the accuracy of described structures. Future work might include additional modeling of these real-valued bond distances.

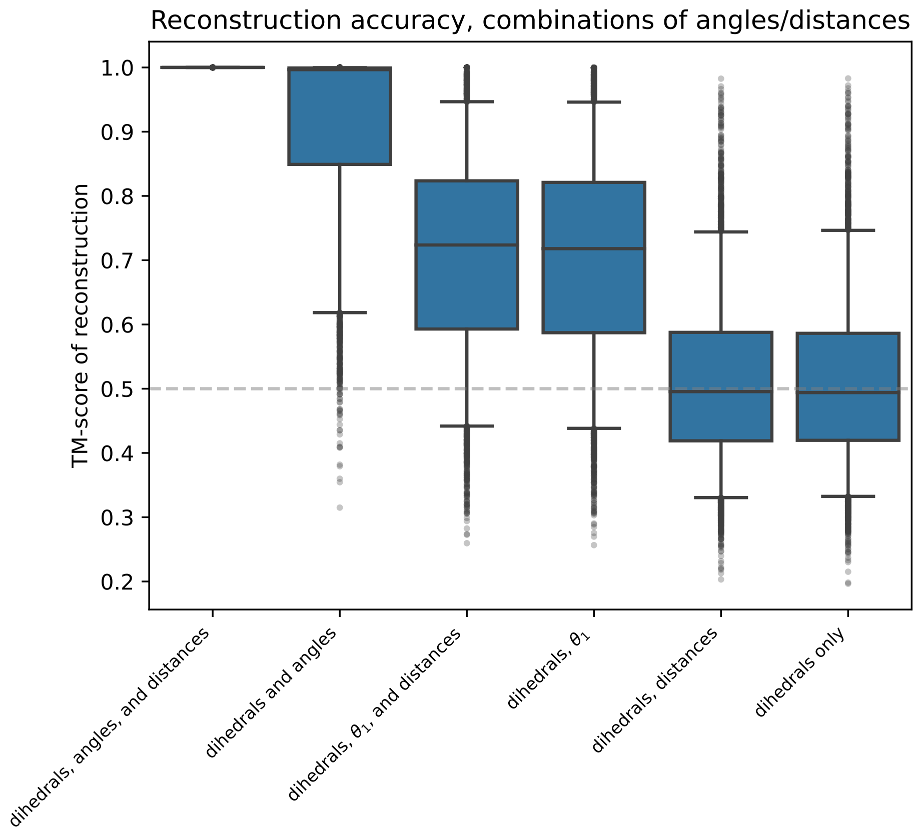

Figure S1: **Various combinations of angles and distances and their ability to reconstruct protein backbones.** A TM-score of 0.5 (dashed grey line) indicates the minimum similarity for two structures to be considered to have the same general shape. Box indicates quartiles of data, with center bar indicating the median. Whiskers indicate 5th and 95th percentile of data; values outside this range are shown as dots. Using all bond angles, dihedral angles, and bond distances perfectly reconstructs Cartesian coordinates from internal angles (first column). The second column corresponds to the formulation used in the main text, where we model the 3 dihedrals and 3 bond angles, but keep the 3 bond distances fixed to average values. Other columns fix even more values to their respective means and result in reconstruction TM scores that are too low to be reliably useful.

One detail when converting between a N-residue set of Cartesian coordinates to a set of $N-1$ angles between consecutive residues is that the latter representation does not capture the first residue's information (as there is no prior residue to orient against). To solve this, we use a fixed set of coordinates to seed all generation of Cartesian coordinates, using the $N-1$ specified angles to build out from this fixed point. For all generations, this fixed point is extracted from the coordinates of the $N - C_\alpha - C$ atoms in the first residue on the N-terminus of the PDB structure 1CRN [78]. Doing so does not result in any meaningful reconstruction error in natural structures.

Effect of length on structure reconstruction

One of the primary concerns of using an angle-based formulation is that small errors might propagate across many residues to culminate in a large difference in overall structure. To try and quantify the effect of this, we evaluate the "lossiness" of the representation itself, and the ability of the model to learn long-range angle dependencies.

We start by evaluating the accuracy of our representation itself over different structure lengths. To do this, we sample 5000 structures of varying length from the CATH dataset. For each, we compare the original 3D coordinate representation x_c and the 3D coordinates obtained after converting the structure to angles and back to coordinates $\hat{x}_c$ using the TM score algorithm, i.e., $\text{TMscore}(x_c, \hat{x}_c)$. We find that longer structures exhibit greater TM score divergences when converted through our representation (Figure S2). However, even at our maximum considered structure length of 128 residues, structures still retain a reconstruction TMscore ≈ 0.9, which is well above the accepted threshold of 0.5 denoting the same fold. This indicates that while our representation itself is slightly lossy, the losses do not change the overall fold. Even when converting longer structures up to 512 residues in length to an angular representation and back, the reconstructed structures still share a TMscore similarity much greater than 0.5 (graph not shown), which suggests that our method could scale up to larger structures. Note, however, that our generative FoldingDiff model is currently only trained on structures up to 128 residues in length – this analysis focuses solely on the potential for errors introduced by our angular representation, and not FoldingDiff's ability to work effectively within this formulation or to design structures longer than 128 residues in length.

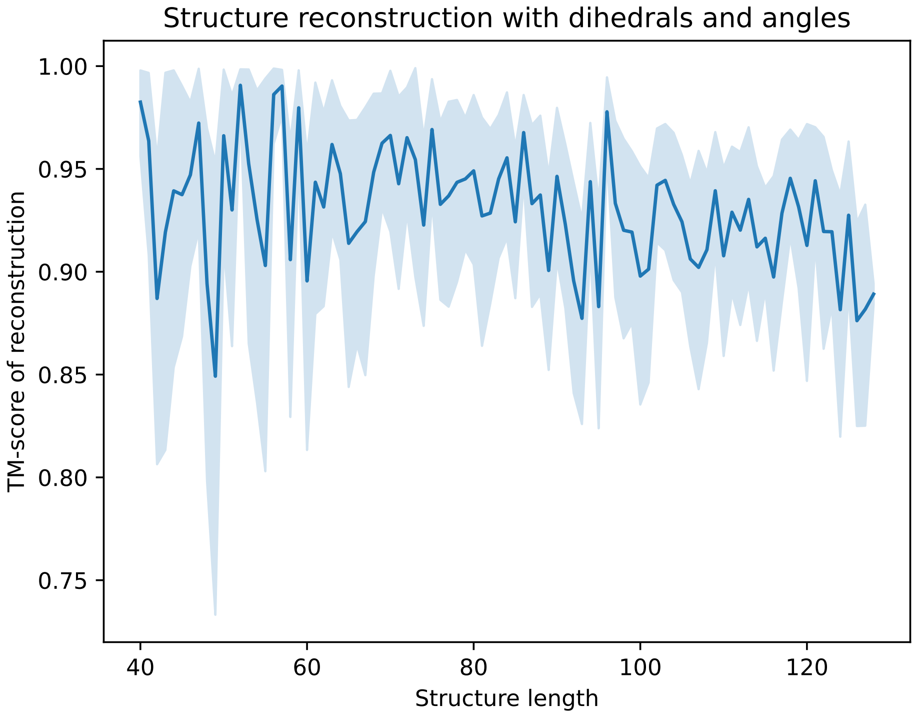

Figure S2: **Faithfulness of reconstruction with dihedrals and angles.** Reconstruction fidelity measured via TMscore (y-axis) when using the 3 dihedrals and 3 angles described in Table 1 and keeping bond distances fixed to average values, evaluated across 5000 structures of varying length (x-axis). Solid line indicates mean value at each length, with shaded region indicating 95% confidence interval. We observe a significant negative correlation between length and reconstruction TM score (Spearman's correlation $\rho = -0.18, p = 7.2 \times 10^{-39}, n = 5000$).

Next, we evaluate our model's ability to successfully reconstruct sequences of varying length. For each structure in our held-out test set ($n = 3040$), we add 250, 100, or 10 timesteps of noise to that structure's angles (recall that we use a total of $T = 1000$ timesteps). We then apply our trained model to these partially noised examples, running them for the requisite remaining itera-

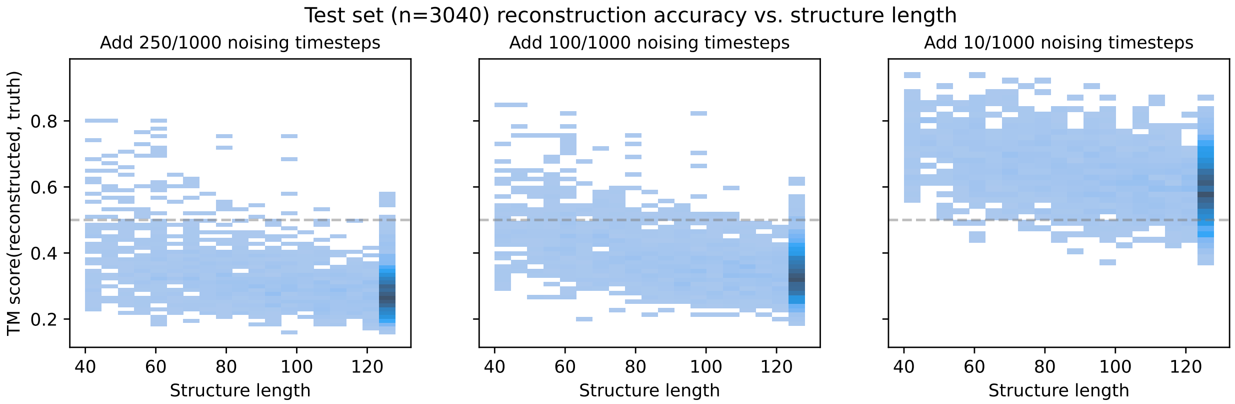

Figure S3: **Test set structure reconstruction accuracy after injecting varying timesteps of noise into angles.** This is evaluated by taking the TM score between the structure specified by the angles reconstructed (i.e., at $T = 0$) after adding 250 (left panel), 100 (middle panel), or 10 (right panel) steps of noise, and the structure specified by the ground truth angles. The x-axis denotes length of the test set structure, and each bar denotes the distribution of TM scores of reconstructed structures (y-axis) within that length. Dotted gray line indicates TM score threshold of 0.5.

tions to fully denoise and reconstruct the angles. Afterwards, we assess reconstruction accuracy by taking the TMscore between the structure specified by the reconstructed angles, and the true structure specified by the original ground truth angles. This attempts to capture the variation in final structures that FoldingDiff can arrive at when starting from partially-noised examples containing varying degrees of information about what the final structure might be. Figure S2 illustrates the relationship between test set structure length and this reconstruction TM score. We find a significant negative correlation between length and reconstruction similarity regardless of noising timesteps added ($t = 250$ Spearman's $\rho = -0.328, p = 2.62 \times 10^{-77}$; $t = 100$ Spearman's $\rho = -0.371, p = 7.71 \times 10^{-100}$; $t = 10$ Spearman's $\rho = -0.370, p = 3.40 \times 10^{-99}$). Notably however, in cases where short structures are reconstructed consistently (i.e., adding 10 timesteps of noise, whereby 100% of 40-residue structures are reconstructed with TMscore ≥ 0.5), the majority of long structures are successfully reconstructed as well (88.1% of 128-residue structures reconstructed with TMscore ≥ 0.5), indicating that there is sufficient signal in the partially noised structure to largely determine the final target structure. This suggests that although generating longer structures is more difficult, FoldingDiff can still handle these structures well.

All in all, these results indicate that while there are certainly challenges with scaling to longer and longer structures, our representation and FoldingDiff itself are nonetheless relatively robust to error accumulation.

Lever arm effects

A key concern when adopting an angular description of protein structures is the potential for single-angle errors to drastically alter the global structure – a lever arm effect. To test this empirically, we take a CATH structure's angular representation a, and then perturb one instance of its constituent dihedral angles (i.e., ϕ, ψ, ω in our main text) by a small amount, thus creating $\hat{a}$. We then calculate the TMscore between the structures specified by a and $\hat{a}$ (Figure S4). We perform a set of perturbations proportional to natural angular variance, and a set of perturbations proportional to angular

differences introduced by FoldingDiff's reconstruction of partially noised test set structures (250 steps, Figure S3).

We repeat this analysis for three randomly selected CATH structures, perturbing each of the three dihedrals for each position in the set of $N-1$ angles specifying the backbone (Figure S4). Despite a and $\hat{a}$ having only one change between them, we observe that even single errors proportional to the native data distribution (Figure S4, left column) can have an appreciable impact on overall structure, especially when they occur in the center of the protein. In comparison, perturbations proportional to FoldingDiff's observed distribution of reconstruction differences result in much less drastic changes in overall structure (Figure S4, right column), suggesting that FoldingDiff is relatively robust to these lever arm effects at this error scale. Nonetheless, we still observe the same trend as with larger perturbations, namely that, controlling for magnitude, errors occurring in central positions have the greatest effects on overall structure.

Atomic clashes

Another concern when adopting an angular view of protein structures is atomic clashes, which can arise when a series of angles generated by FoldingDiff results in molecules that clash in Cartesian space. To understand how often these occur, we analyze clash frequencies for three sets of proteins: natural CATH structures between 50 and 128 residues in length, FoldingDiff generations, and RFDiffusion [18] generations. Both RFDiffusion and FoldingDiff were asked to unconditionally generate 10 structures for each length in the range $l \in [50, 128)$ for a total of 780 structures each. For this analysis, we consider two non-consecutive backbone atoms with Van der Waal radii x, y to be clashing if their pairwise Euclidean distance is less than $0.63(x + y)$, indicating a large overlapping volume. Distances for Van der Waal radii are taken from Bondi [79]. The frequency of clashes and median clashes per structure are shown in Table S1. Although FoldingDiff's raw generations exhibit significantly more clashes than both natural CATH structures and RFDiffusion's generations, these can be easily filtered out or structurally refined, as we discuss below.

Structural relaxation

FoldingDiff's raw generations can be passed through structural refinement methods to resolve clashes, taking inspiration from previous works [13]. To do this, we take each backbone generated by FoldingDiff and use ProteinMPNN to sample 8 different amino acid sequences predicted to fold to that backbone (this step and the sequences generated are shared with the designability analyses in the main text). We then use FASPR [80] to pack the side chains specified by each amino acid sequence onto the original backbone, yielding a total of 8 candidate all-atom structural models corresponding to the original backbone. We then apply PyRosetta's [81] FastRelax constrained relaxation protocol to each of these 8 candidate models and choose the relaxed structure with the highest TMscore to the original backbone as the final representative all-atom structure.

Comparing these representative all-atom structure models to original generations from FoldingDiff, we find that 455/780 generations have a TMscore $\geq$ 0.5 before and after attaching side chains with relaxation – i.e., most structures retained their overall fold. Furthermore, among these 455 structures, 433/455 (95.2%) are clash-free. Across all 780 relaxed structures, 733/780 (94.0%) are clash-free. Together, these values indicate that side chain packing and subsequent relaxation

effectively adds complete side chain orientations and resolves structural clashes while typically retaining the overall original generated structure. We also find that this relaxation TM score is highly correlated with scTM designability (Figure S5, Spearman's $\rho = 0.65, p = 8.61 \times 10^{-96}$), suggesting that side chain packing followed by structural relaxation may serve as a computationally faster and more robust (as it involves combining fewer deep learning models) method for evaluating quality of generated structures.

Table S1: Number of atomic clashes observed within CATH structures, FoldingDiff generations, and RFDiffusion generations of comparable length. The two rows for FoldingDiff correspond to structures that are directly generated without relaxation or refinement ("w/o relax") and structures that have been structurally refined that also retain meaningful similarity to the original generation ("relaxed").

Category	Total structures	Clash-free structures	Median clashes per structure
CATH	13963	13720 (98.3%)	0
RFDiffusion [18]	780	768 (98.5%)	0
FoldingDiff (w/o relax)	780	364 (46.7%)	2
FoldingDiff (relaxed)	455	433 (95.2%)	0

Substituting Cartesian coordinates in place of angular formulation

We perform an ablation of our internal angle representation by replacing our framing of proteins as a series of inter-residue internal angles with a simple Cartesian representation of C_α coordinates $x \in \mathbb{R}^{N \times 3}$. Notably, this Cartesian representation is no longer rotation or shift invariant. We train a denoising diffusion model with this Cartesian representation, using the same variance schedule, transformer backbone architecture, and loss function, but sampling from a standard Gaussian and with all usages of our wrapping function w removed. This represents the same modelling approach as our main diffusion model, with only our internal angle formulation removed.

To evaluate the quality of this Cartesian-based diffusion model's generated structures, we calculate the pairwise distances between all C_α atoms in its generated structures and compare these with distance matrices calculated for real proteins and for our internal angle diffusion model's generations. For a real protein, this produces a pattern that reveals close proximity between pairwise residues where the protein is folded inwards to produce a compact, coherent structure (Figure S6a). However, similarly visualizing the C_α pairwise distances in the Cartesian model's generated structures yields no significant proximity or patterns between any residues (Figure S6b). This suggests that the ablated Cartesian model cannot learn to generate meaningful structure, and instead generates a nondescript point cloud. Our internal angle model, on the other hand, produces a visualization that is very similar to that of real proteins (Figure S6c). Simply put, our model's performance drastically degrades when we change only how inputs are represented. This demonstrates the importance and effectiveness of our internal angle formulation.

Consistency of results across seeds and variation in scTM pipelines

We evaluate generations from FoldingDiff using a range of random initial seeds, in addition to the generation results reported in the primary text. For each of five additional random seeds, we generate 10 different structures for each length $l \in [50, 128)$ yielding a total of 780 generated

backbones per seed, all produced from the same trained model. For each set of generated backbones, we compute scTM scores using ProteinMPNN for inverse folding and OmegaFold for fold prediction. Each generation run produces a consistently high number of designable structures. We additional perform secondary structure annotation and find that each generation run produces structures containing a realistic mixture of secondary structure elements (Figure S7). Jointly, these results demonstrate that FoldingDiff is able to produce high-quality structures consistently, regardless of random seed.

Addition to consistency across random seeds, we also evaluate how consistent our results are when applying different inverse folding methods for predicting amino acid sequences for FoldingDiff's generated backbones – specifically substituting ESM-IF1 [65] in place of ProteinMPNN, keeping all other aspects of the scTM evaluation procedure the same. Designability using ESM-IF1 is reported in Table S3. Overall, we find that a high percentage of our structures are still designable, but ESM-IF1 results in significantly poorer overall designability than ProteinMPNN.

Table S2: **Self-consistency TM scores (using ProteinMPNN and OmegaFold) across random seed replicates**. Short structures are defined as having 70 residues or fewer ($n = 210$); long structures are defined as having more than 70 residues ($n = 570$). Values from our main text are reproduced in the last row for ease of reference.

Random seed	Designable	Designable, short ($n = 210$)	Designable, long ($n = 570$)
1	173	72	101
2	154	75	79
3	185	90	95
4	182	86	96
5	187	76	111
7344 (main text)	177	80	97

Table S3: **Self-consistency TM scores calculated using ESM-IF1 to perform inverse folding, rather than ProteinMPNN.** These analyze the same FoldingDiff generations as Table S2, and are likewise folded with OmegaFold. ESM-IF1 consistently results in much lower scTM scores ($p = 0.031$, two-sided Wilcoxon signed-rank test).

Random seed	Designable	Designable, short ($n = 210$)	Designable, long ($n = 570$)
1	117	61	56
2	105	64	41
3	122	76	46
4	115	72	43
5	126	67	59
7344 (main text)	111	57	54

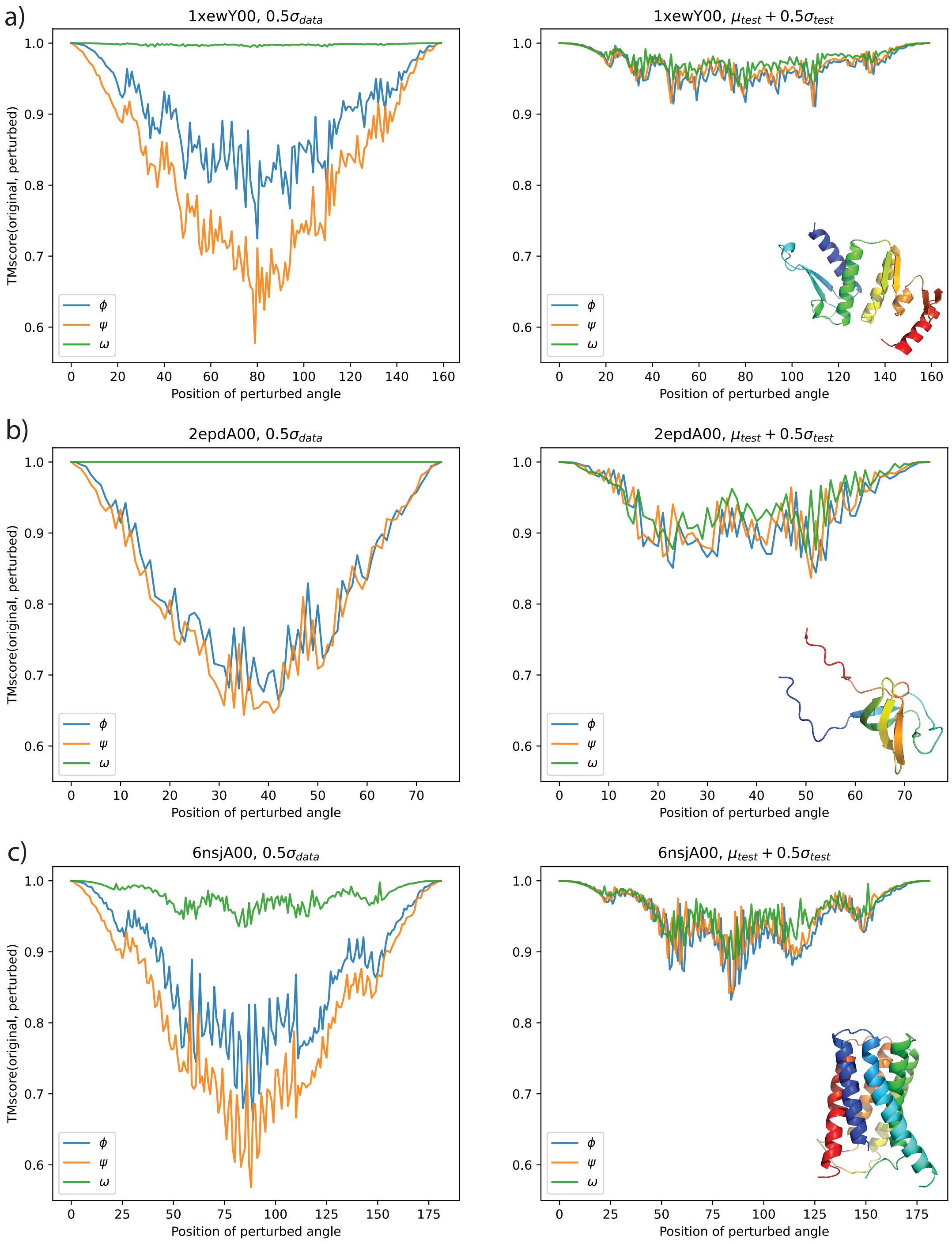

Figure S4: **Empirical demonstration of lever arm effects on three CATH structures.** For each of three randomly selected CATH structures (a-c), TMscore comparison of the "correct" structure specified by the original series of six angles describing protein backbones (see Table 1), to a perturbed structure specified by a set of angles including a single perturbed dihedral (ϕ, ψ, ω; blue, orange, green lines respectively). The relevant dihedral was perturbed by either 0.5 times its natural standard deviation ($0.5\sigma_{\text{data}}$; left column), or 0.5 times the standard deviation in angular difference arising when FoldingDiff reconstructs that angle from partially noised test structures ($0.5\sigma_{\text{test}}$), offset by the average difference ($\mu_{\text{test}} + 0.5\sigma_{\text{test}}$; right column). This perturbation was applied to each of the $N - 1$ positions along the structure, for each of the 3 dihedral backbone torsional angles. Each plot shows the change in TMscore (y-axis) as a function of the perturbed dihedral (colored lines) and the position of the perturbation (x-axis).

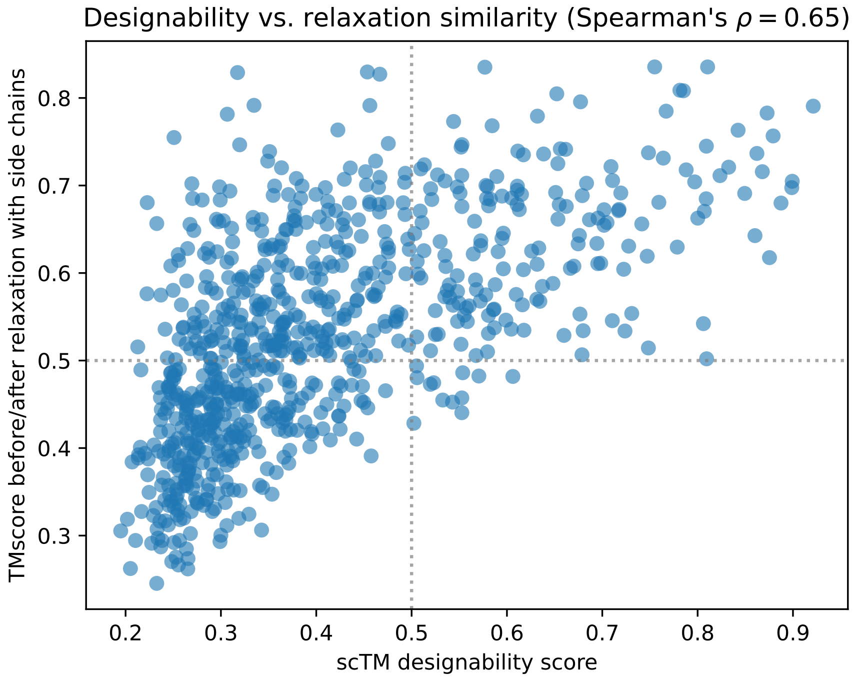

Figure S5: **Relationship between scTM and relaxation TM score.** For each of the 780 structures generated and evaluated in the main text, a relaxation TM score was computed between the original backbone structure and the relaxed all-atom structure containing packed side chains. The amino acid sequences used for scTM analysis were considered, and rather than folding the sequences, FASPR [80] and PyRosetta [81] were used to pack the corresponding side chains directly onto FoldingDiff's generated backbone. After structural relaxation, the packed structure most similar to the original generation was used to calculate a relaxation TM score (y-axis) relative to the original backbone. There is a significant positive correlation between designability and relaxation TM score (Spearman's $\rho = 0.65, p = 8.61 \times 10^{-96}, n = 780$).

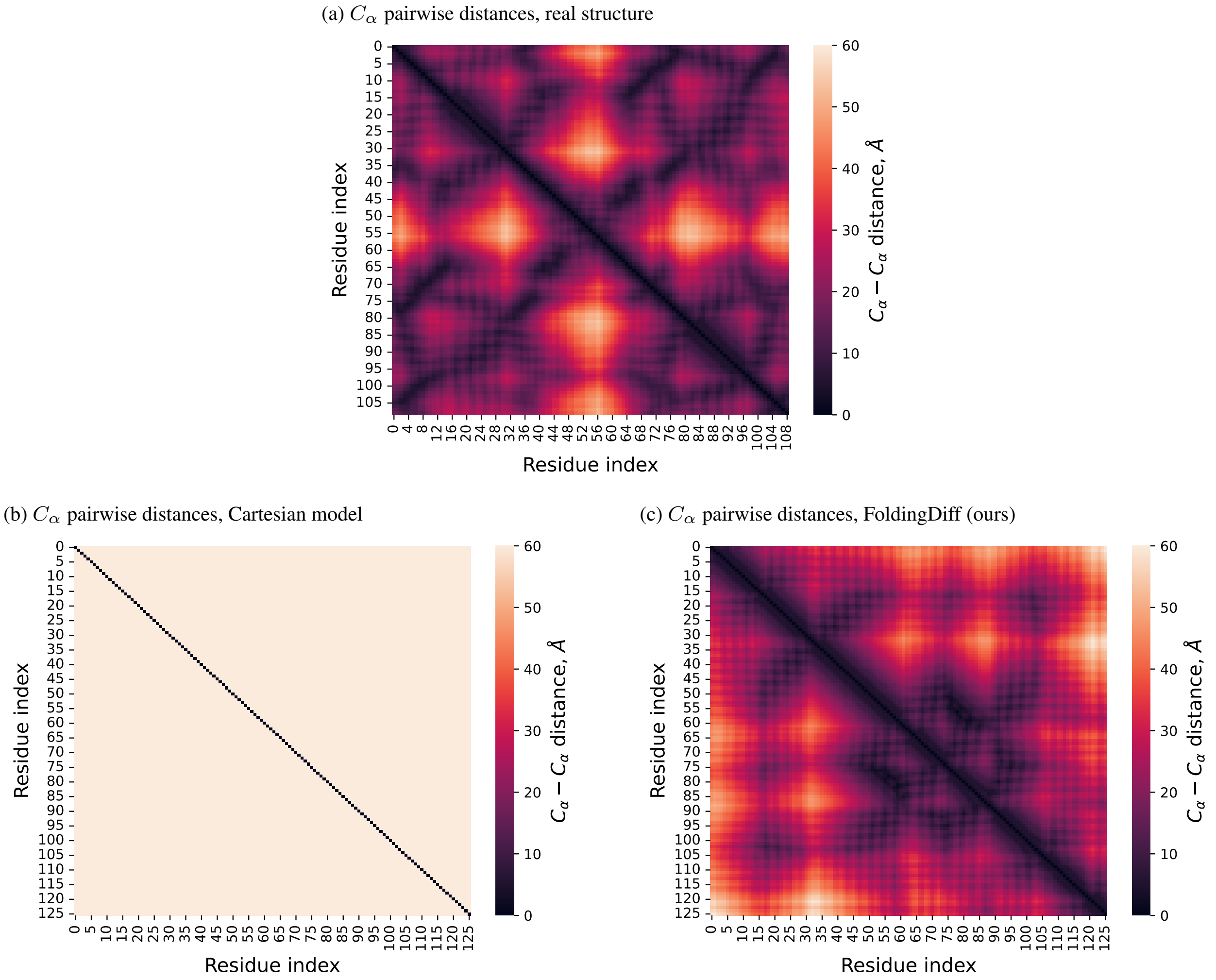

Figure S6: **Pairwise distances between all C_α atoms in various protein backbone structures, all of similar length.** All panels use the same color scale. **(a)** Visualization of a set of distances for a real protein structure; note the visual patterns that correspond to various secondary structures and potential contacts and interactions between residues. **(b)** Visualization of these distances for a structure generated by an ablated model that replaces our proposed internal angle representation with Cartesian coordinates, which results in no coherent structural generation. **(c)** Visualization of these distances for generated structures from our FoldingDiff model. These structures compactly fold to create many potential contacts, just as real proteins do.

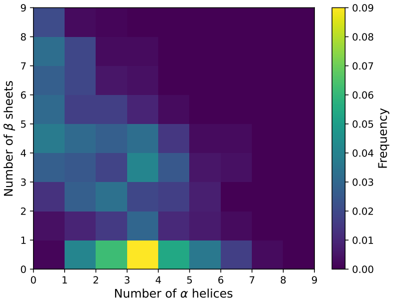

(a) Generations, seed 1

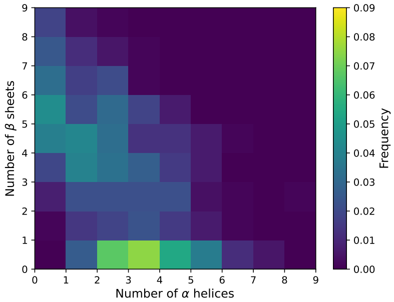

(b) Generations, seed 2

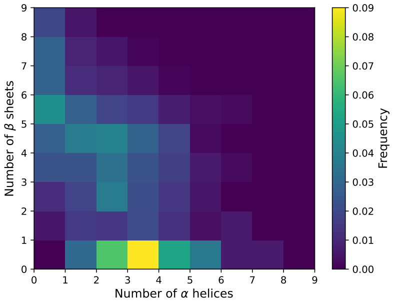

(c) Generations, seed 3

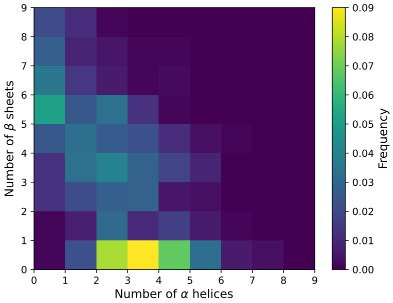

(d) Generations, seed 4

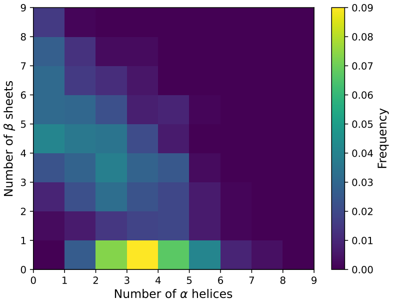

(e) Generations, seed 5

Figure S7: **Secondary structure occurrence in independent generation replicates.** a-e For each of the 5 replicates shown in Table S2, we use P-SEA to annotate secondary structures. Each run's generations contain a mixture of α helices (x-axis) and β sheets (y-axis) that is comparable to natural structures (Figure 3b). FoldingDiff generates reasonable, complex structures consistently.

Additional Supplementary Figures

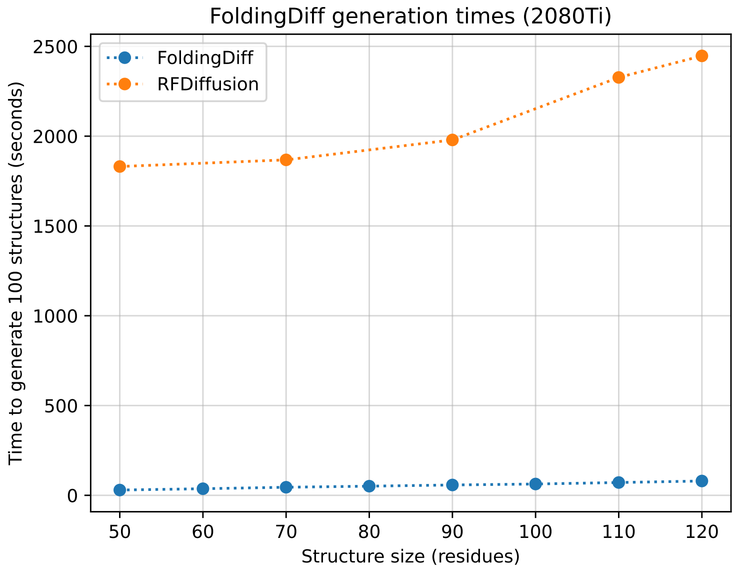

Figure S8: **FoldingDiff generation time.** Time (y-axis) for FoldingDiff (blue) and RFDiffusion [18] (latest version as of September 2023, orange) to unconditionally generate 100 structures of varying length (x-axis) on a machine with an Intel i9-9960X processor and a single NVIDIA 2080Ti GPU. Both methods leverage GPU acceleration for this benchmark, and are run using out-of-the-box settings. FoldingDiff's runtime grows approximately linearly with respect to size of the generated structure; generating 100 structures of 120 residues each takes about 80 seconds. RFDiffusion is slower and exhibits runtime scaling superlinear with respect to structure size; generating 100 structures of 120 residues each takes approximately 41 minutes.

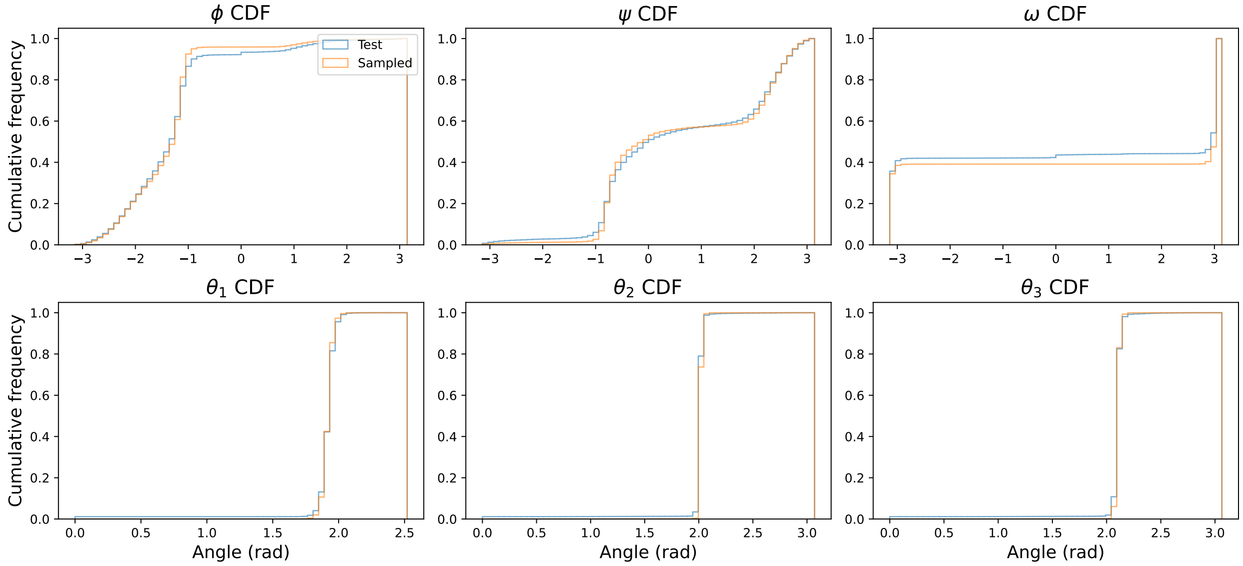

Figure S9: **Comparison of the cumulative distribution functions (CDF) of angular values in test set (blue) and generated samples (orange).** Top row shows dihedral angles (torsional angles involving 4 atoms), and bottom row shows bond angles (involving 3 atoms). Figure 2b shows the histogram distributions corresponding to these CDFs.

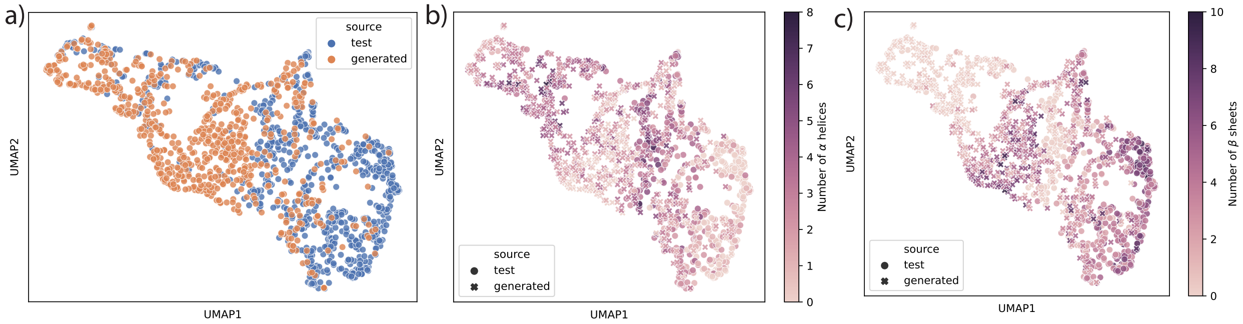

Figure S10: **Visualization of Gauss integral embeddings and structural features of FoldingDiff generations and natural CATH structures.** (a) UMAP visualization of Gauss integral embeddings for natural test-set ($n = 847$, blue) structures between 50 and 128 residues in length, and structures generated by FoldingDiff ($n = 780$, orange). These same embeddings are additionally colored by the number of α helices (b) and β sheets (c) as detected by P-SEA. In panels (b-c), marker types denote whether each point corresponds to a CATH (circle) or FoldingDiff (cross) example.

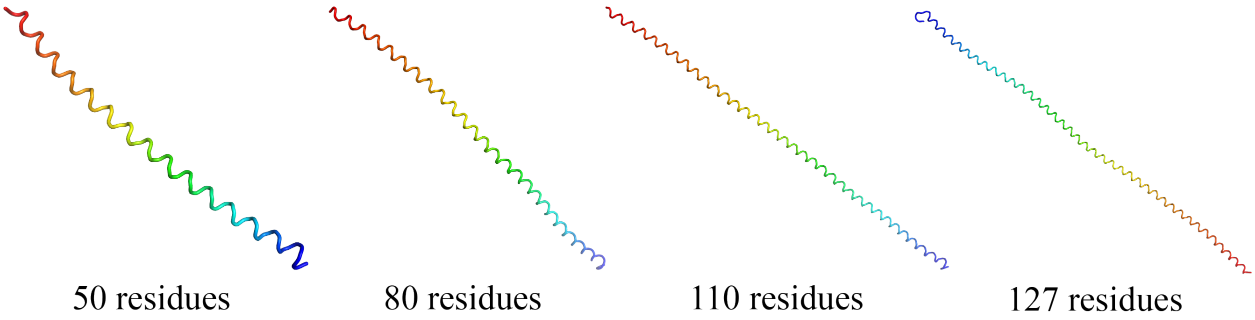

Figure S11: **Structures generated using an autoregressive (AR) baseline approach trained on the same angle-based formulation we propose.** This approach predicts the next set of angles given all prior angles, and can be thus used to iteratively generate structures in an autoregressive fashion (see Supplementary Information for additional details). However, the structures generated this way are all straight α helices, regardless of initial prompt angles (see Figure S12a). This complete lack of diversity and meaningful complexity indicates that while this AR model can produce technically correct structures, it cannot be used for generative modeling to any meaningful capacity. Figure 4d analogously illustrates FoldingDiff's generations, which are structurally much more diverse.

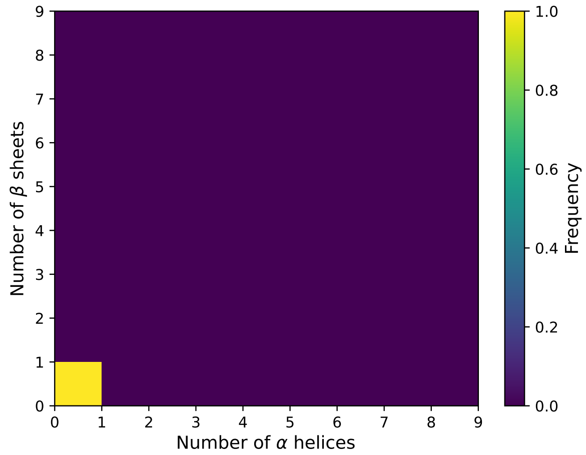

(a) AR baseline, secondary structure co-occurrence

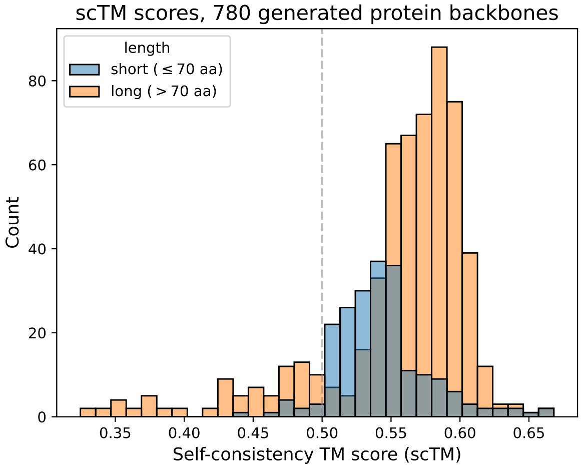

(b) AR baseline, scTM designability

Figure S12: **Secondary structure elements (a) and designability (b) for structures generated by the autoregressive baseline model.** We observe that using P-SEA to annotate these generated structures detects exclusively singular α helices, and no β sheets (S12a). This quantifies the observations in Figure S11 that the AR model has collapsed into repeatedly generating these coils. We find that these helices exhibit greater designability via scTM scores (computed using ProteinMPNN and OmegaFold) (S12b), with 693 of the 780 structures having scTM $\geq$ 0.5, though this increase is not meaningful due to the utter lack of diversity in generated sequences.

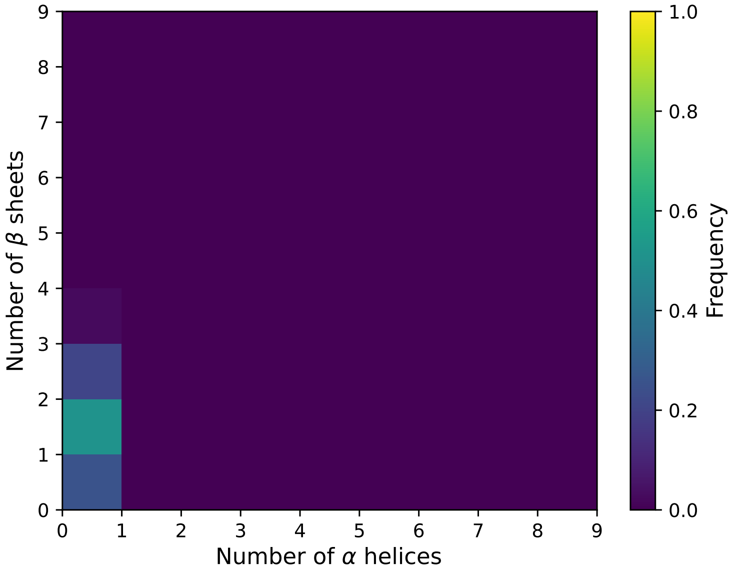

Figure S13: **Secondary structure co-occurrence 2D histogram for protein backbones generated by randomly shuffling angle sets found in natural proteins.** This sampling strategy preserves the overall distribution of angles while disrupting the correct ordering that produces reasonable overall structures. Secondary structure elements are annotated using P-SEA. There are no alpha helices detected, and only a handful of sheets that like arose from random chance.

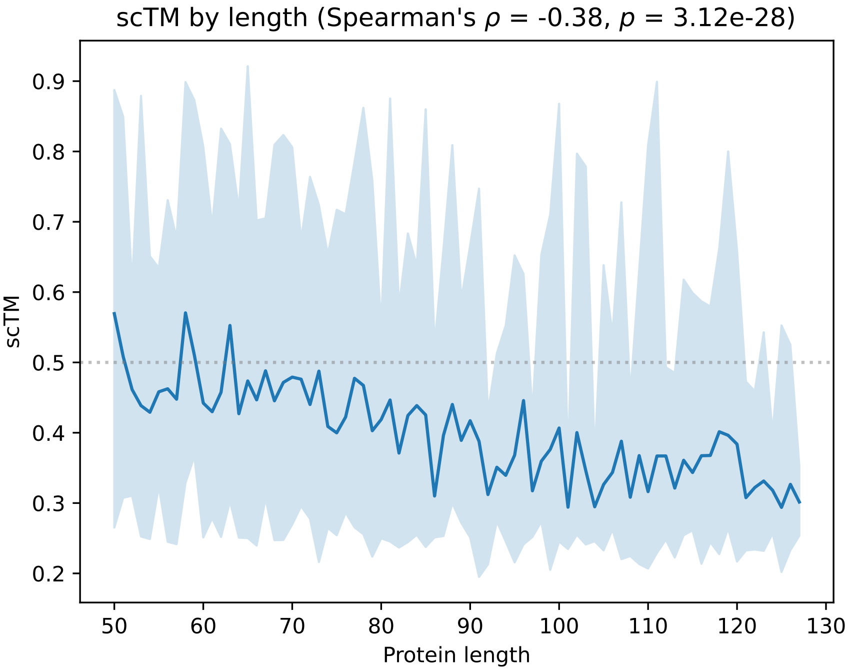

Figure S14: **Distribution of scTM scores by generated structure length.** Self-consistency TM score (scTM, y-axis), evaluated with ProteinMPNN [47] OmegaFold [48], versus length (x-axis) for all of FoldingDiff's generated proteins. The solid line indicates the average scTM at each length, with the shaded region indicating range between the highest and lowest observed scTM for that length. Dotted gray horizontal line indicates scTM cutoff for designability at 0.5. There is a significant correlation between the two, with longer proteins exhibiting lower designability scTM scores (Spearman's $\rho = -0.38$, $p = 3.12 \times 10^{-28}$, $n = 780$).

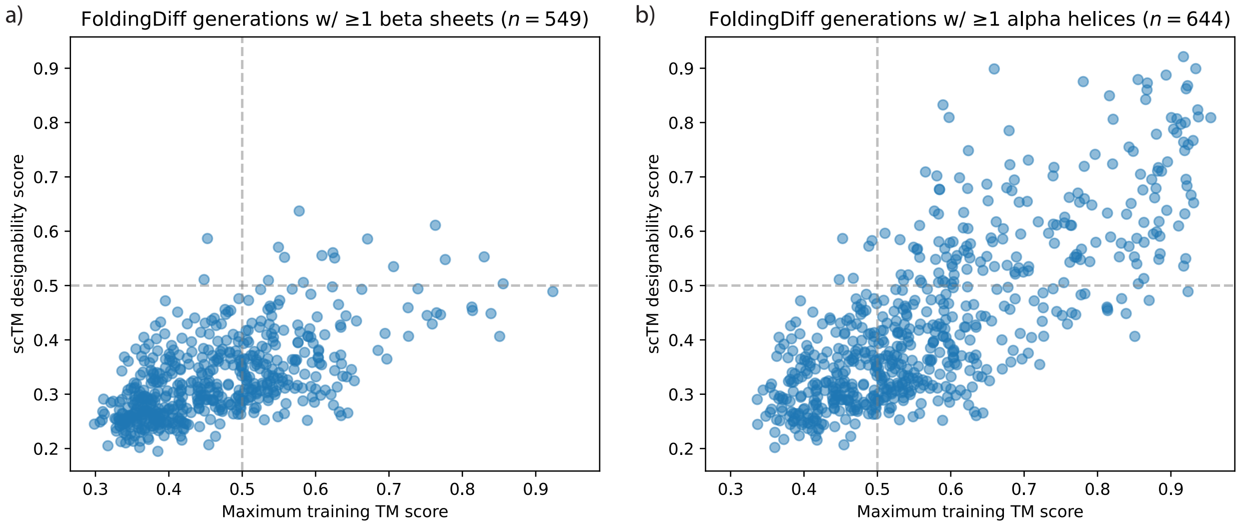

Figure S15: **scTM designability scores broken down by secondary structure content.** Relationship between training TM score similarity (x-axis) and scTM designability (y-axis) for FoldingDiff generated structures with at least one beta sheet (a) and with at least one alpha helix (b). Structures generated by FoldingDiff (Figure 4, $n = 780$) are separated into two categories corresponding to (a) whether or not they contain at least one beta sheet (panel a, $n = 549$), and (b) whether not they contain at least one alpha helix (panel b, $n = 644$). These categories are non-exclusive – i.e., structures with at least one beta sheet may also contain alpha helices and vice versa.

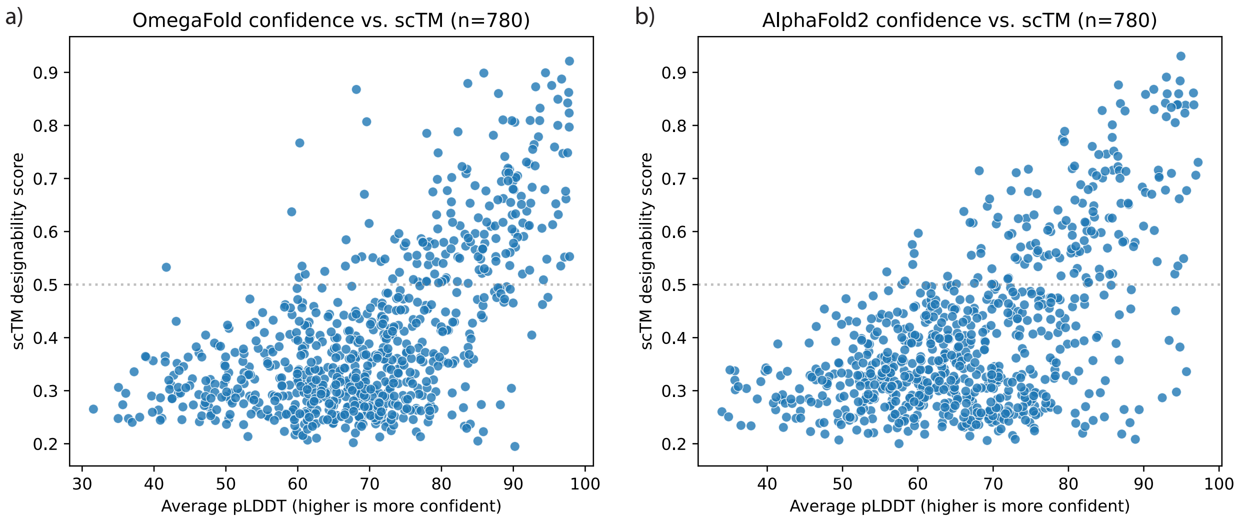

Figure S16: **scTM designability scores for FoldingDiff's generations as a function of protein folder confidence.** For each structure generated by FoldingDiff, ProteinMPNN [47] was used to sample 8 candidate amino acid sequences likely to realize the generated structure and OmegaFold [48] (a) or AlphaFold2 [51] (b) was then used to computationally fold and validate these sequences. The scTM designability score (y-axis) was computed using the best match among the 8 candidates. Both OmegaFold (a) and AlphaFold2 (b) also produce predicted local-distance difference test (pLDDT) scores per residue, indicating confidence in their predicted folds. The average pLDDT (x-axis) for the closest-matching predicted structure for each of FoldingDiff's generations (i.e., the structure yielding scTM scores) was compared to scTM (y-axis). There is a significant positive correlation between pLDDT and scTM for both OmegaFold (Spearman's $\rho = 0.56, p = 4.61 \times 10^{-65}, n = 780$) and AlphaFold2 (Spearman's $\rho = 0.47, p = 1.42 \times 10^{-44}, n = 780$). Dotted horizontal line indicates 0.5 scTM threshold for designability.

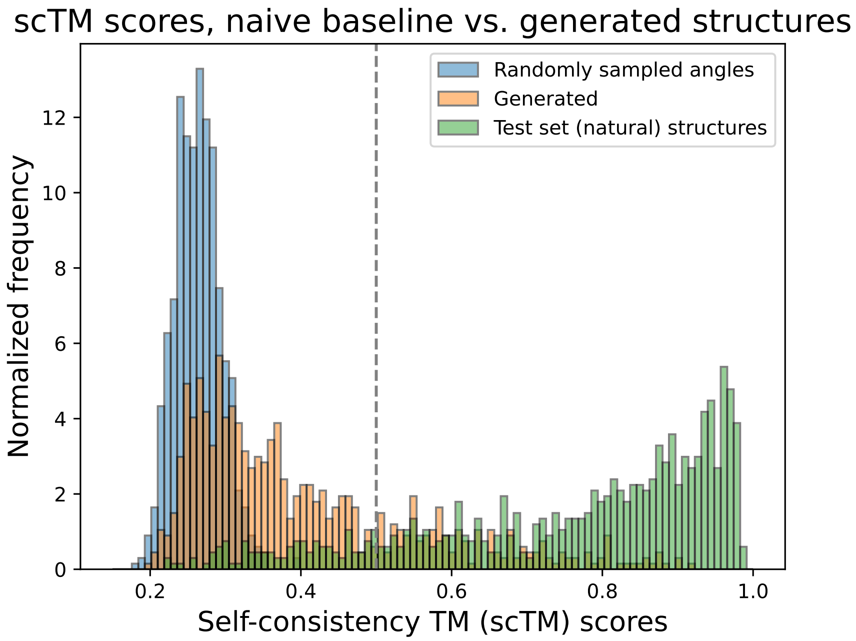

Figure S17: **Distribution of scTM scores for our generated structures (orange), compared to scTM scores for structures created by randomly shuffling naturally-occurring internal angles (blue).** The randomly sampled angles result in no designable structures, despite perfectly capturing the overall distribution and pairwise relations between angles. This suggests our method correctly learns the spatial ordering of angles that folds a valid structure. We additionally take a set of 780 experimental structures and pass them through our scTM pipeline to evaluate the fragility of this pipeline itself. We find that 87% of natural proteins (green) are designable; this forms a soft upper bound for what would be achievable by sampling from the true data distribution.

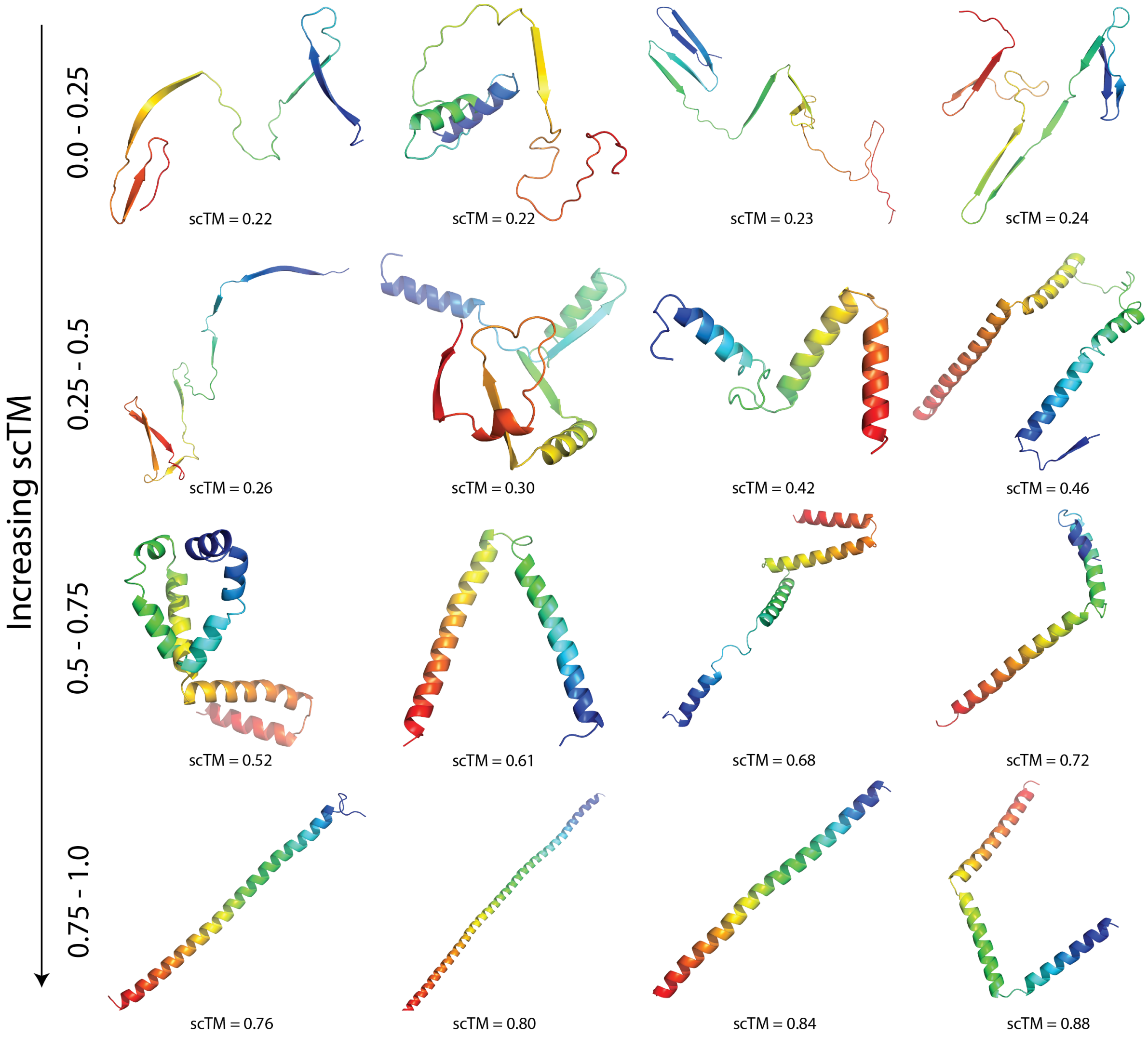

Figure S18: **Representative FoldingDiff generations spanning the range of scTM designability scores.** From top to bottom, each row shows structures of increasing designability with the ranges $[0.0, 0.25), [0.25, 0.5), [0.5, 0.75), [0.75, 1.0)$. Within each row, structures are sorted in increasing designability from left to right. Structures are colored in a rainbow hue from N to C terminus.

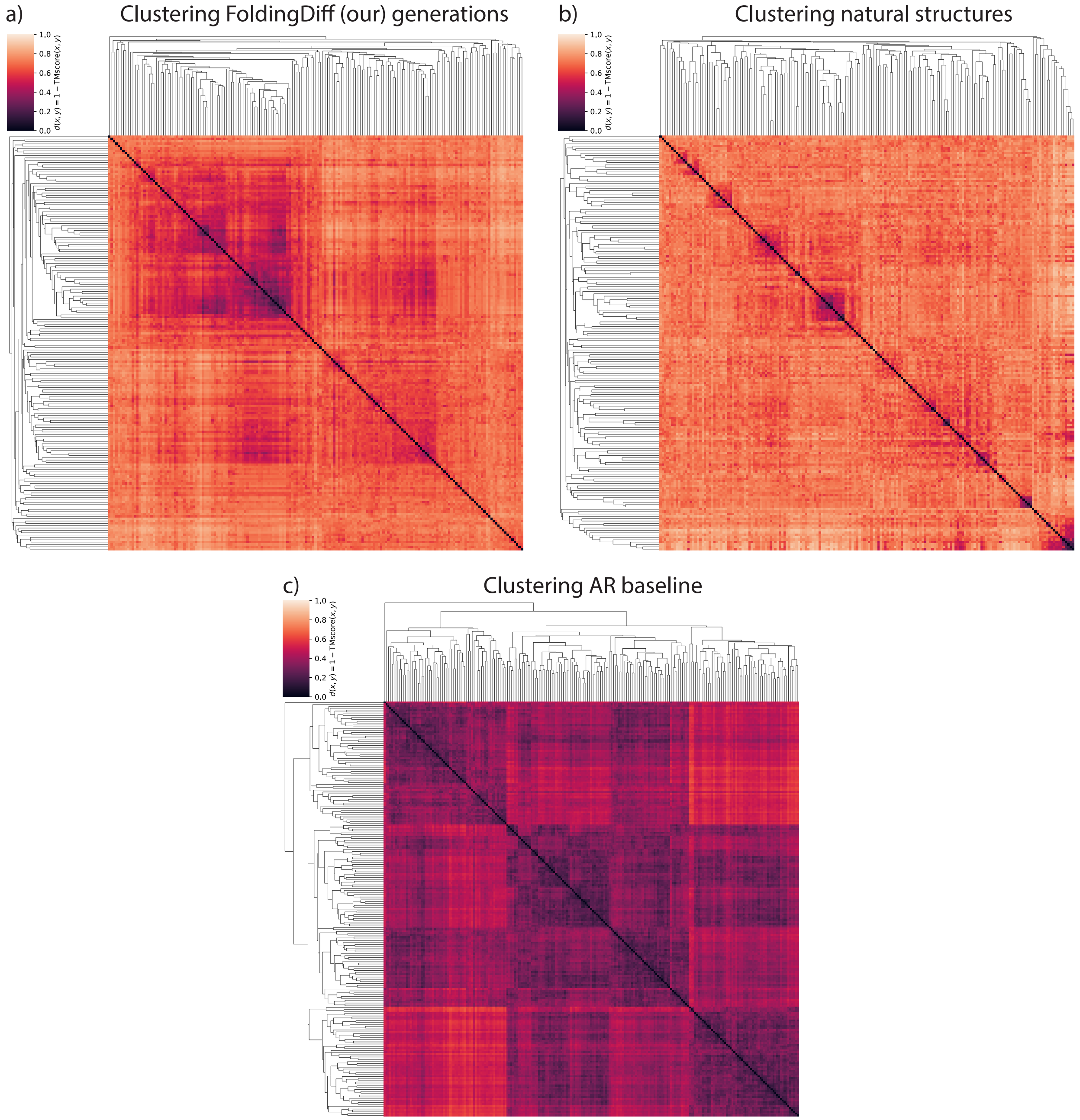

Figure S19: **Diversity spanned by FoldingDiff's generated designable backbones, compared to various baselines structure sets.** Across all analyses, we use hierarchical clustering using $1 - \text{TMscore}(x, y)$ as a pairwise distance metric. Color scheme is consistent across all visualizations, with darker shades indicating more similar structures within the heatmap. Visualization and clustering of FoldingDiff's generations, filtering for scTM ≥ 0.5 **(a)**, a randomly selected set of naturally-occurring protein backbones **(b)**, and generations from the autoregressive baseline **(c)**.
