# Supplementary material for: Protein structure generation via folding diffusion
Source: Nat Commun. 2024 Feb 5;15:1059. doi: 10.1038/s41467-024-45051-2 (PMC10844308; doi:10.1038/s41467-024-45051-2)
Supplement: Supplementary file 3 — Reporting Summary [file 41467_2024_45051_MOESM3_ESM.pdf]

nature portfolio

Corresponding author(s): Ava P. Amini

Last updated by author(s): Dec 18, 2023

Reporting Summary

Nature Portfolio wishes to improve the reproducibility of the work that we publish. This form provides structure for consistency and transparency in reporting. For further information on Nature Portfolio policies, see our Editorial Policies and the Editorial Policy Checklist.

Statistics

For all statistical analyses, confirm that the following items are present in the figure legend, table legend, main text, or Methods section.

n/a	Confirmed	
☐	☒	The exact sample size (*n*) for each experimental group/condition, given as a discrete number and unit of measurement
☒	☐	A statement on whether measurements were taken from distinct samples or whether the same sample was measured repeatedly
☐	☒	The statistical test(s) used AND whether they are one- or two-sided<br>*Only common tests should be described solely by name; describe more complex techniques in the Methods section.*
☒	☐	A description of all covariates tested
☒	☐	A description of any assumptions or corrections, such as tests of normality and adjustment for multiple comparisons
☐	☒	A full description of the statistical parameters including central tendency (e.g. means) or other basic estimates (e.g. regression coefficient) AND variation (e.g. standard deviation) or associated estimates of uncertainty (e.g. confidence intervals)
☐	☒	For null hypothesis testing, the test statistic (e.g. *F*, *t*, *r*) with confidence intervals, effect sizes, degrees of freedom and *P* value noted<br>*Give P values as exact values whenever suitable.*
☒	☐	For Bayesian analysis, information on the choice of priors and Markov chain Monte Carlo settings
☒	☐	For hierarchical and complex designs, identification of the appropriate level for tests and full reporting of outcomes
☐	☒	Estimates of effect sizes (e.g. Cohen's *d*, Pearson's *r*), indicating how they were calculated

Our web collection on statistics for biologists contains articles on many of the points above.

Software and code

Policy information about availability of computer code

Data collection	FoldingDiff is trained publicly available protein domain structures from the CATH S40 database, version 4.3.0. This is available at https://www.cathdb.info/ and we additionally provide a copy at Zenodo DOI 10.5281/zenodo.8388270 (https://zenodo.org/records/8388270).
Data analysis	Modeling and data analysis were done in Python (version 3.8) and various open source packages. These notably include PyTorch (version 1.12), PyTorch Lightning (version 1.6.4), BioTite (version 0.34), scikit-learn (version 1.2.1), numpy (version 1.22.3), and pandas (version 1.1.5). Plotting was done within matplotlib and seaborn; proteins are visualized using PyMOL (version 2.5.4). An exhaustive list of required software packages and versions are available within the environment.yml file in the publicly available code repository.

For manuscripts utilizing custom algorithms or software that are central to the research but not yet described in published literature, software must be made available to editors and reviewers. We strongly encourage code deposition in a community repository (e.g. GitHub). See the Nature Portfolio guidelines for submitting code & software for further information.

Data

Policy information about availability of data

All manuscripts must include a data availability statement. This statement should provide the following information, where applicable:

- Accession codes, unique identifiers, or web links for publicly available datasets
- A description of any restrictions on data availability
- For clinical datasets or third party data, please ensure that the statement adheres to our policy

FoldingDiff is trained publicly available protein domain structures from the CATH S40 database, version 4.3.0. This is available at https://www.cathdb.info/ and we additionally provide a copy at Zenodo DOI 10.5281/zenodo.8388270 (https://zenodo.org/records/8388270). We also reference the PDB structure 1CRN (https://doi.org/10.2210/pdb1CRN/pdb). All generated structures (n=780) that we discuss in our main text, as well as metadata annotations describing each structure, are available at Zenodo DOI 10.5281/zenodo.8388286 (https://zenodo.org/records/8388286).

Research involving human participants, their data, or biological material

Policy information about studies with human participants or human data. See also policy information about sex, gender (identity/presentation), and sexual orientation and race, ethnicity and racism.

Reporting on sex and gender	N/A; no human participants or data.
Reporting on race, ethnicity, or other socially relevant groupings	N/A; no human participants or data.
Population characteristics	N/A; no human participants or data.
Recruitment	N/A; no human participants or data.
Ethics oversight	N/A; no human participants or data.

Note that full information on the approval of the study protocol must also be provided in the manuscript.

Field-specific reporting

Please select the one below that is the best fit for your research. If you are not sure, read the appropriate sections before making your selection.

☒ Life sciences ☐ Behavioural & social sciences ☐ Ecological, evolutionary & environmental sciences

For a reference copy of the document with all sections, see nature.com/documents/nr-reporting-summary-flat.pdf

Life sciences study design

All studies must disclose on these points even when the disclosure is negative.

Sample size	Sample sizes (i.e., number of generated structures to evaluate) were chosen to match prior computational works that similarly generated protein backbones, such as to facilitate comparisons.
Data exclusions	All generated protein backbones were analyzed, and no exclusion or filtering criteria were applied. At the training stage, protein domains fewer than 40 amino acids long were excluded from training, as these are less likely to include rich secondary structure motifs to train the model to subsequently produce rich structures.
Replication	Generation results were repeated with 5 different random seeds in addition to the set of generated structures presented in the main text. These additional rounds of generation were analyzed for their designability, as was done in the main text; these results are summarized in Table S2. We find that the proportion of generated structures that are designable is highly consistent across these replicates, indicating that FoldingDiff is consistent in its ability to generate high quality structures.
Randomization	N/A; since all our analysis is done at the level of individual protein structures, and we are not working with any living systems, it is not necessary to randomize for underlying confounding variables.
Blinding	N/A; our work focuses generating new protein backbone structures, and we use unbiased two-sided statistics to objectively measure how similar or different our generations are compared to various baseline methods and natural structures. As there is no human element in how different proteins are "treated", these two-sided tests are sufficient to establish the presence of a statistically significant difference.

Reporting for specific materials, systems and methods

We require information from authors about some types of materials, experimental systems and methods used in many studies. Here, indicate whether each material, system or method listed is relevant to your study. If you are not sure if a list item applies to your research, read the appropriate section before selecting a response.

Materials & experimental systems

n/a	Involved in the study
☒	☐ Antibodies
☒	☐ Eukaryotic cell lines
☒	☐ Palaeontology and archaeology
☒	☐ Animals and other organisms
☒	☐ Clinical data
☒	☐ Dual use research of concern
☒	☐ Plants

Methods

n/a	Involved in the study
☒	☐ ChIP-seq
☒	☐ Flow cytometry
☒	☐ MRI-based neuroimaging

Plants

Seed stocks	*Report on the source of all seed stocks or other plant material used. If applicable, state the seed stock centre and catalogue number. If plant specimens were collected from the field, describe the collection location, date and sampling procedures.*
Novel plant genotypes	*Describe the methods by which all novel plant genotypes were produced. This includes those generated by transgenic approaches, gene editing, chemical/radiation-based mutagenesis and hybridization. For transgenic lines, describe the transformation method, the number of independent lines analyzed and the generation upon which experiments were performed. For gene-edited lines, describe the editor used, the endogenous sequence targeted for editing, the targeting guide RNA sequence (if applicable) and how the editor was applied.*
Authentication	*Describe any authentication procedures for each seed stock used or novel genotype generated. Describe any experiments used to assess the effect of a mutation and, where applicable, how potential secondary effects (e.g. second site T-DNA insertions, mosiacism, off-target gene editing) were examined.*
